# The Effectiveness of a Community Psychiatric Rehabilitation Program Led by Laypeople in China: A Randomized Controlled Pilot Study

**DOI:** 10.3389/fpsyt.2021.671217

**Published:** 2021-11-15

**Authors:** Ying Chen, Chow S. Lam, Hong Deng, Eva Yau, Kam ying Ko

**Affiliations:** ^1^Mental Health Center, West China Hospital, Sichuan University, Chengdu, China; ^2^Department of Psychology, Illinois Institute of Technology, Chicago, IL, United States; ^3^Departement of Psychiatry, University of Hong Kong, Hong Kong SAR, China; ^4^Hong Kong Youth Foundation, Hong Kong SAR, China

**Keywords:** community-based, psychiatric rehabilitation, multidisciplinary teams, mental illness, randomized-control

## Abstract

**Background:** Community psychiatric rehabilitation has proven effective in supporting individuals and their families in recovering from mental illness. The delivery of evidence-based community rehabilitation services, however, requires health care workers to possess a set of specially trained knowledge and skills. Most developing countries, including China, do not have specially trained mental health personnel. The purpose of this study was to test the feasibility and efficacy of a community psychiatric rehabilitation program delivered by laypeople.

**Method:** We conducted a randomized controlled study. Patients at two sites in Chengdu, China, were randomly assigned to either the laypeople-delivered (LPD) community psychiatric rehabilitation group (*N* = 49) or the drop-in center control group (*N* = 45). The outcomes were changes in symptoms, social functioning, and family functioning over 6 months, as measured by the Positive and Negative Syndrome Scale (PANSS), the Personal and Social Performance Scale (PSP), the Family Burden Scale of Disease (FBS), and the Family APGAR index.

**Results:** The number of sessions received over the 12-week period of treatment ranged from 20 to 100%, with a mean completion rate of 77.32% for all 12 sessions. Statistically significant interactions between group and time were found for the total PANSS [*F*_(2, 94)_ = 12.51, *p* < 0.001] and both the Negative PANSS [*F*_(2, 94)_ = 5.89, *p* < 0.01] and Positive PANSS [*F*_(2, 94)_ = 6.65, *p* < 0.01] as well as the PSP [*F*_(2, 94)_ = 3.34, *p* < 0.05], FBS [*F*_(2, 94)_ = 5.10, *p* < 0.01], and Family APGAR index [*F*_(2, 94)_ = 4.58, *p* < 0.01]. The results showed that the experimental group outperformed their counterparts in symptom management, personal social functioning, family care burden, and coherence.

**Conclusion:** These results support the feasibility and efficacy of having laypeople deliver psychiatric rehabilitation services. A discussion and limitations of the study have been included.

## Introduction

Psychiatric rehabilitation, also known as psychosocial rehabilitation, has changed service delivery for people with severe and persistent mental illness. It catalyzed the deinstitutionalization movement in America in the early 1970s, moving psychiatric treatment from institution based to community based. With accumulating evidence, community psychiatric rehabilitation has become the main service delivery model. However, in Asia, due to a lack of resources and specialized trained rehabilitation professionals, community psychiatric rehabilitation is still in the early stages of development (1, 2). Tse, Huang, and Zhu (2), in addressing Asian mental health care reforms, pointed out that China has a population of 1.3 billion, with an estimate of 173 million Chinese citizens suffering from diagnosable mental disorders, of whom 158 million have never received any treatment (3). Approximately 16 million Chinese citizens have severe mental illness, and this figure is expected to grow. Most of these individuals go without treatment due to a lack of community rehabilitation resources (4). The insufficient number of mental health professionals exacerbates mental health care problems. Presently, China has only 4,000 fully qualified, licensed psychiatrists (5) and has few professionally trained allied health workers, such as rehabilitation counselors, social workers, occupational therapists, and rehabilitation psychologists, to work with people with severe persistent mental illness.

*In Asian countries, about 70% of clients with schizophrenia live with their family. The unpredictable and bizarre behaviors of the patients, stressors of stigma upon the family, and family conflicts in the caring process have greatly impacted the daily lives of caregivers. Meanwhile, the preparation and knowledge about disorders of caregivers might influence the client outcome* *(**6**)*. Community psychiatric rehabilitation has proven effective in supporting individuals and their families recovering from mental illness (6–9).

In many Western countries, clinical and social services for people with schizophrenia are coordinated by specialist community-based multidisciplinary teams. However, such specialist services are not presently feasible in low-income and developing countries because of serious human and financial resource constraints. Hence, the development of alternative methods for the provision of accessible, community-based services for people with schizophrenia within these countries is a global public health priority (10). In most developing countries, including China, alternative methods are needed for the provision of community-based psychiatric rehabilitation. “Task sharing” (11), a widely adopted strategy, has been used by developing countries to address the shortage of qualified mental health workers. The strategy uses lay health workers with appropriate training and supervision to provide access to evidence-based mental health care interventions. Thus, the current pilot study sought to accomplish two goals. The primary aim of the study was to assess the feasibility of a laypeople-delivered (LPD) community psychiatric rehabilitation program. The second goal was to assess whether the LPD community psychiatric rehabilitation program confers greater benefit than the control condition for the patient's social functioning and the family's psychological well-being.

## Methods

### Design

We used a randomized controlled design to compare the efficacy of LPD community psychiatric rehabilitation to that of a community drop-in center control group. Participants were randomized to receive either LPD community psychiatric rehabilitation (a 12-week program) or drop-in center services (control group). Assessments were conducted at baseline, posttreatment (3 months), and follow-up (6 months) by trained research assistants.

### Participants

This study was approved by the research ethics committee of West China Hospital of Sichuan University, written informed consent was obtained from the parents/guardians of all participants, and consent was obtained from the study participants prior to participation. Participants were recruited from two different organizations in Chengdu, a major city in Sichuan, China. The West China Hospital Mental Health Center, a major mental hospital in Chengdu, provided a list of possible participants who had been discharged from the hospital from August 1, 2012, to July 31, 2014. The Yulin Community Health Center provided a list of residents in the community who had a diagnosis of mental disorders. Each potential participant was interviewed with the Structured Clinical Interview for Diagnostic and Statistical Manual of Mental Disorders, Fourth Edition (SCID), for their eligibility for the study by psychiatrists or graduate students who were trained in SCID. After informed consent was obtained from each eligible participant, the participants were randomly assigned to either the LPD community psychiatric rehabilitation group or the drop-in center control group, and a baseline assessment was conducted.

G^*^Power 3.1 was used to calculate the required sample size with an effect size of 0.25 (medium), alpha error probability of 0.05, and power of 0.80. For repeated-measures ANOVAs with between- and within-group interactions, the required sample size was 62. In view of possible dropouts and incomplete attendance of the program, we recruited 108 participants with a major diagnosis of schizophrenia for the study. Admission criteria for the study included (1) having a diagnosis of schizophrenia or schizoaffective disorder; (2) being between 16 and 60 years of age; and (3) not having a diagnosis of mental incompetence or organic brain syndrome or a primary diagnosis of substance dependence.

We used SPSS software to randomize the participants; 58 participants from 108 participants were randomly assigned to the LPD group. Ninety-four participants completed the entire course of the study. Nine participants, seven due to <25% attendance and two due to refusal, dropped out of the LPD community psychiatric rehabilitation group, and five participants from the control group refused to continue in the study due to transportation problems. The dropouts did not differ from the rest of the sample in terms of characteristics or functions. Thus, the LPD community psychiatric rehabilitation group consisted of 49 participants, and the control group had 45 participants.

### Assessment Measures

#### Psychiatric Symptom Severity

A Chinese version of the PANSS (12) was used to measure psychiatric symptom severity. It is a structured clinical interview consisting of 30 items designed to assess the severity of symptoms over the past week on a 7-point scale (1 = absent to 7 = extreme); higher scores indicate more severe symptoms. The PANSS raters were trained to an interrater agreement of 80% on a series of videotapes for which “gold standard” consensus ratings had been determined by a group of experienced raters. The PANSS subscales were used to measure negative symptoms, positive symptoms, and dysphoric mood. The reported psychometric properties of the PANSS include Cronbach's alpha coefficients of 0.73 on the positive scale, 0.83 on the negative scale, and 0.87 on the general psychopathology scale.

#### Social Functioning

A Chinese version of the Personal and Social Performance Scale (PSP) (13) was used to assess the participant's social functioning. The PSP was developed based on the social functioning component of the DSM-IV social and occupational functioning assessment scale (SOFAS). The scale assesses four main areas of social functioning: socially useful activities; personal and social relationships; self-care; and disturbing and aggressive behaviors. Difficulty in each area is rated on a six-point scale (absent, mild, manifest, marked, severe, or very severe), with lower ratings indicating better social functioning. A global item ranging from 1 to 100 in 10-point intervals is rated by the interviewer, where lower scores indicate worse functioning. Cronbach's alpha of 0.84 was reported.

#### Family Functioning

A Chinese version of the FBS was used to assess the family burden (14). The FBS has 24 items spread across six factors: economic burden, impact on daily activities, impact on social life, impact on free time, impact on physical health, and impact on mental health. The ratings of 24 items are made with a three-level scale from 0 to 2, with higher scores indicating a greater burden. Cronbach's alpha coefficient was 0.87, and split-half reliability was 0.94 for FBS.

A Chinese version of the Family APGAR index was used to measure family function (15). The Family APGAR scale scores five dimensions of family function: adaptability, partnership, growth, affection, and resolution. The scores of the scale assess overall satisfaction with family life and provide a composite measure of perceived family functioning. The total score ranges from 0 to 20. The higher the score, the higher the level of perceived family function. Cronbach's alpha of 0.86 was reported.

### Treatments

#### Development of the LPD Community Psychiatric Rehabilitation Program

Several strategies were used in the development of the LPD community psychiatric rehabilitation program, namely, literature review, expert consultation, and group discussion. Through an extensive literature review, we have identified several models that would be relevant to the current pilot project. These models include illness management and recovery (7), case management (16), psychosocial rehabilitation (17), and family psychoeducation (18, 19). With input from our consultant (CL) and several discussion meetings among the community psychiatric rehabilitation team members (the authors of the article), the structure and contents of the LPD community psychiatric rehabilitation program were formed. Underlying practice principles of our LPD community psychiatric rehabilitation program were drawn from several lines of behavioral science research, which found that people are more apt to change when they are in the context of a positive relationship, when they set their own goals, are taught skills, receive support, have positive expectations or hope for the future and when they believe in their self-efficacy (9, 17, 20). All of these change elements demonstrated in the behavioral science research literature became critical ingredients for the LPD community psychiatric rehabilitation services.

With these guiding principles, we identified core components of the LPD community psychiatric rehabilitation program. These core components include psychoeducation provided to the patients and their families (about mental illness, its treatment, and recovery), medication management (using cognitive-behavioral approaches to enhance medication adherence), case management (developing a SMART goal-oriented recovery plan), social skills training (strengthening social support and community reintegration), stress management training (for the management of stress and persistent symptoms), coping and problem solving training (using counseling, cognitive-behavioral therapy [CBT], and problem-solving skills to deal with personal issues and problems that would interfere with the recovery plan). Table 1 outlines the modules and contents of the LPD community psychiatric rehabilitation program.

**Table 1:** Overview of the topics for the LPD community psychiatric rehabilitation program modules.

**Module**	**Topic**	**Goals**	**# of 60-min sessions**
1	Facts about mental illnesses	• Etiology of schizophrenia, a brain disease • Identify symptoms associated with schizophrenia • Dispel myths about schizophrenia • Address stigma, public and self stigmas	2
2	Family psychoeducation	• Education about serious mental illnesses • Information resources, especially during periods of crises • Skills training and ongoing guidance about managing mental illnesses • Problem solving • Social and emotional support	8
3	Recovery and rehabilitation	• Understand the process of recovery and rehabilitation • Increase awareness of recovery • Help clients become aware of people with schizophrenia who lead productive lives	2
4	Medication management	• Discuss benefits and side effects of medications • Help clients weigh the pros and cons of taking medications • Teach behavioral skills tailored to facilitate medication adherence	4
5	Social skills training	• Basic conversation skills and getting closer to people, making eye contact, starting and ending a conversation, making and refusing requests, expressing opinions to others, and showing appropriate emotions • Working on correcting deficits in receptive, processing, and sending social skills • Teach strategies for increasing support, such as making friends and finding places to meet people • Discuss how building social support can facilitate recovery	8
5	Stress management	• Explain that stress and biological vulnerability causes the symptoms of schizophrenia • Discuss strategies for reducing stress and biological vulnerability • The relationship between stress thoughts (automatic negative thoughts), emotions and behavior, cognitive restructuring and mindfulness, and relaxation and breathing techniques • Healthy and unhealthy stress coping methods	8
6	Case management	• Set personal recovery goals • Develop SMART goals • Help consumers and families problem-solve issues related to the treatment plan • Case review and modification	12
7	Coping and problem solving	• Teach the patient to cope with problems and persistent symptoms • Teach a problem-solving model • Help clients identify common problems and symptoms that cause distress • Practice coping strategies for persistent symptoms	8

#### Treatment Protocol

**Figure 1:**
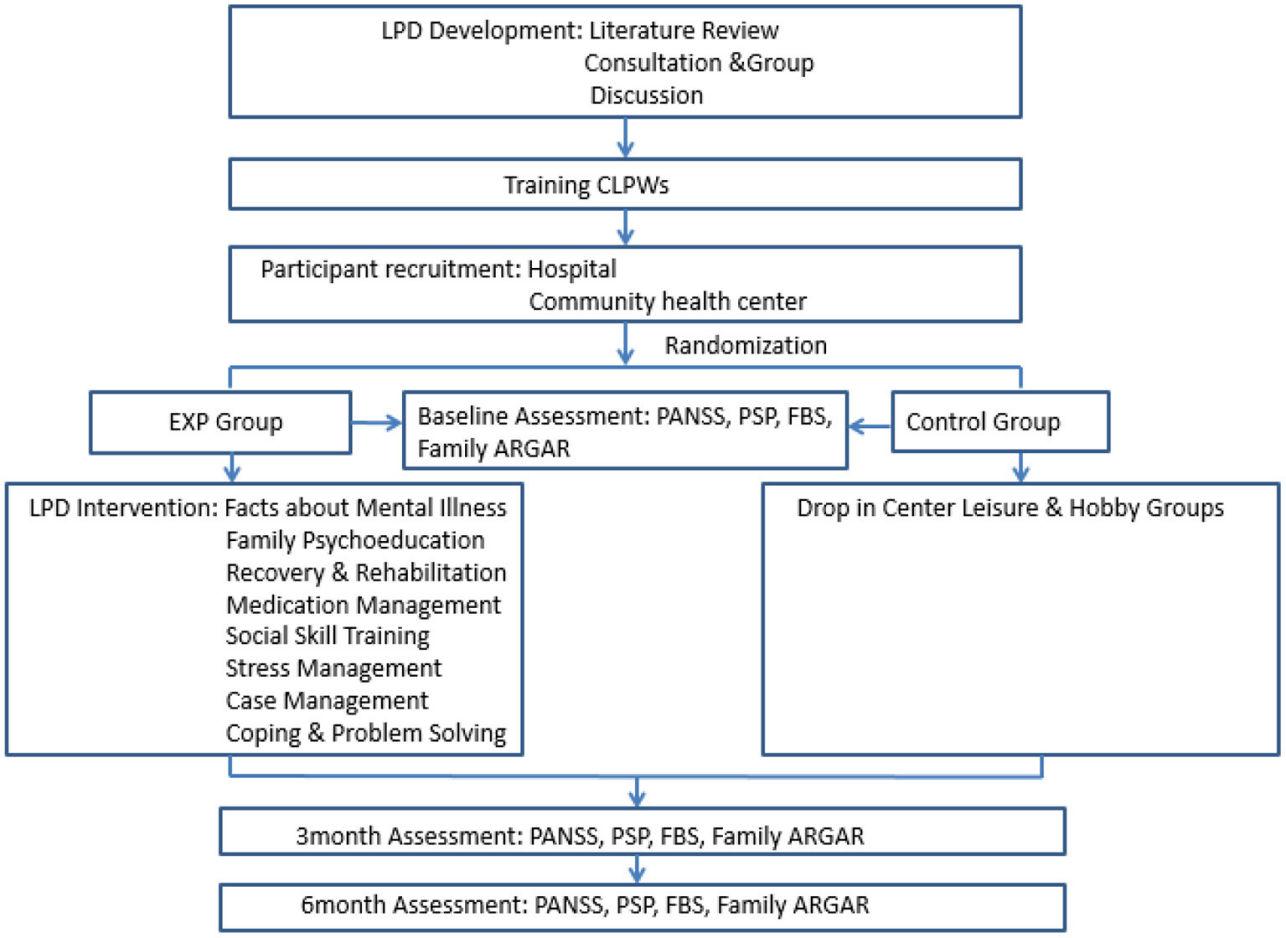
Flowchart of the study (LPD psychiatric rehabilitation program).

Figure 1 shows the flowchart of this study. After randomization, the experimental group attended the LPD community psychiatric rehabilitation program modules, as shown in Table 1. The module contents were converted to PowerPoint to assist in the group-based delivery of the curriculum. Every session had the same routine, which meant that the whole program followed a structured pattern. Each module was led by two community lay psychiatric worker (CLPW) instructors who were trained on how to deliver the module curriculum. A combination of educational, motivational, and cognitive-behavioral teaching strategies and homework assignments was used in the delivery of the module. Each session lasted 60 min, meeting once a week. For the family psychoeducation module, both the patients and their family members participated in the session. This group could provide an opportunity to review the fundamentals of illness management with concerned others in a context where clients could obtain support and help in pursuing their personal recovery goals. The module was conducted following the Substance Abuse and Mental Health Services Administration (SAMHSA) guidelines.

The case management module was conducted on an individual basis. This module lasted 12 weeks. Each case manager had three to five cases depending on the difficulties of the cases and the availability of the case manager. There were 15 CLPW case managers involved in the study. In addition to working with the participant, the case manager also worked with the family to help participants learn self-management strategies and pursue their personal goals. In case management, the participant's individual goals were often broken down into smaller steps to facilitate continuous progress toward achieving the goals.

#### Drop-in Center Control Group

The drop-in center, a part of the community psychiatric rehabilitation program, provided a place for participants to get together to engage in various leisure and hobby activities, such as singing, painting, listening to music, arts and crafts, and local outings. It was available to both experimental and control group participants. The drop-in center opened 3 days a week to the community from 9:00 a.m. to 4:00 p.m., and attendance was voluntary.

#### Training of Lay People in Community Psychiatric Rehabilitation

CLPWs were recruited locally from the West China Hospital Mental Health Center and local volunteer organizations. CLPWs must have completed high school education, preferably with a college degree and with no prior or little training in mental health or psychiatric rehabilitation. We recruited 12 CLPWs from community volunteer organizations. Many of them had served as disaster volunteers during the 2008 earthquake in Sichuan. Fifteen nurses from the West China Hospital Mental Health Center who had no formal training in psychiatric rehabilitation also served as CLPWs. All CLPWs were women with an average of 14.6 years of education. Half of the CLPWs had basic counseling skills training through the 2008 earthquake relief project. The CLPWs were responsible for delivering the community psychiatric rehabilitation program. Psychiatric professionals (two psychologists from the United States, two psychiatrists from China, and one occupational therapist from Hong Kong) supervised the CLPWs and were responsible for the overall development and implementation of the intervention.

Under the leadership of the second author (CL), the psychiatric rehabilitation team developed a training curriculum based on the community psychiatric rehabilitation program topics and contents. The CLPW training program was 3 months long. Experts and professionals in psychiatric rehabilitation from overseas, including the United States, Hong Kong, and Singapore, conducted the training. Depending on the content of the modules, a knowledge-based curriculum required 4–8 h of didactic training. Modules on skill-based training, such as social skills, stress management, counseling and cognitive behavioral therapy skills, and case management skills, involve hands-on practice, role playing, and supervision. Each of the skills training modules required 8–16 h. In addition to module training, all case managers received weekly face-to-face or Skype supervision for an hour from the supervising professionals. Competence of the CLPWs was established through observation and the demonstration of content knowledge and skills. All CLPWs met the minimum level of competence.

*During the trial, we kept the research and intervention teams physically separate*.

#### Drug Interventions

Both participants of two groups visited the psychiatric clinic on a regular basis, every 1–3 months a time. *Psychiatric clinics mainly offer drug intervention, and psychiatrists adjust the dose of antipsychotics according to the severity of the patient's symptoms. Family members of 80% of patients will accompany them to the visit*. Medical insurance reimbursed 90% of the patients' medical expenses, and the community subsidy is 1,500 yuan a year.

#### Statistical Analyses

To test the between- and within-group differences, we used repeated-measures ANOVAs on data from baseline, 3 months, and 6 months and complemented these analyses with traditional significance testing. Follow-up analyses were conducted for interaction effects. Effect sizes of the comparisons were reported. The Mauchly sphericity criterion (*W*) was used to judge the validity of the conditions for repeated-measures ANOVAs, and the conditions were met. Multiple stepwise regression models were used to identify the predictor of gaining benefit from the LPD program (Supplementary Materials, statistical analysis).

## Results

### Sample Characteristics

Demographic characteristics and baseline measures are presented in Table 2. Most of the participants had a diagnosis of schizophrenia, and 2% had a diagnosis of schizoaffective disorder. Participants had a mean age of 28.80 years (SD = 10.55) with an onset age of 20.79 (SD = 6.14). Participants were more likely to be male (59%) and had a mean education level of 12.17 years (SD = 2.70). No significant differences were found in any of the sociodemographic variables between the two groups.

**Table 2:** Sample characteristics, total sample.

	**LPD psychiatric rehabilitation (*****N*** **=** **49)**		**Drop-in center control (*****N*** **=** **45)**			
**Variables**	***N***	**%**	***N***	**%**	**t/**χ^2^/*z*/F****	***p***
Age (M ± SD)	29.68 ± 10.81		27.71 ± 10.24		0.931	0.354
Gender						
Male	31	55	28	62	0.484	0.545
Female	25	45	17	38		
Diagnosis						
Schizophrenia	55	98	44	98	2.064	0.154
Schizoaffective	1	2	1	2		
Age of onset (M ± SD)	21.23 ± 6.55		20.24 ± 5.63		0.802	0.425
Duration of illness (mean rank)	52.11		49.62		−0.426	0.67
Number of episodes (mean rank)	52.02		49.73		−0.4	−0.768
Number of hospitalizations (mean rank)	49.08		53.39		0.69	0.443
PANSS^a^ (M ± SD)	57.86 ± 13.92		53.11 ± 14.12		−1.692	0.094
PSP^b^ (M ± SD)	59.64 ± 16.29		64.22 ± 16.17		1.409	0.162

a *PANSS, Positive and Negative Syndrome Scale (possible scores range from 30 to 210, with higher scores indicating more severe symptoms).*

b *PSP, Personal and Social Performance Scale (possible scores range from 1 to 100, with lower scores indicating poorer functioning)*.

### Treatment Participation

The LPD psychiatric rehabilitation group: the number of sessions attended over the 12-week period of treatment ranged from 20–100%, with a mean completion rate of 77.32% for all 12 sessions. Among the participants who completed the baseline assessment, seven participants attended <25% of the program and were considered dropouts and excluded from the analyses.

The drop-in center control group: the participants of the drop-in center attended 6.38 ± 4.36 (1–25) times within the 3 months it was open.

### Evaluation of Treatment Efficacy

Table 3 provides a summary of the mean differences and levels of significance associated with the primary outcome measures from pretreatment to posttreatment. Repeated-measures analyses of variance were conducted to examine the impact of the LPD community psychiatric program with group (intervention vs. control) as the independent variable and assessment point (pretest, 3-month test, or 6-month test) as time. For these analyses, the group-by-time interaction tests whether participants in the LPD community psychiatric program improved more than clients in the drop-in center control group. An analysis was performed for each of the outcome measures, including the two subscales of the PANSS, PSP, FBS, and Family APGAR. Statistically significant interactions between group and time were found for the total PANSS [*F*_(2, 94)_ = 12.51, *p* < 0.001] and both the Negative PANSS [*F*_(2, 94)_ = 5.89, *p* < 0.01] and Positive PANSS [*F*_(2, 94)_ = 6.65, *p* < 0.01] as well as the PSP [*F*_(2, 94)_ = 3.34, *p* < 0.05], FBS [*F*_(2, 94)_ = 5.10, *p* < 0.01], and Family APGAR [*F*_(2, 94)_ = 4.58, *p* < 0.01].

**Table 3:** Means (standard deviations) and effect sizes for outcome variables for each treatment group before and after the intervention.

**Variables**	**LPD community rehabilitation (*****N*** **=** **49)**			**Drop-in center control (*****N*** **=** **45)**			**group** **×** **time**	
	**Preintervention**	**3-month follow-up**	**6-month follow-up**	**Preintervention**	**3-month follow-up**	**6-month follow-up**		
	**M ± SD**	**M ± SD**	**M ± SD**	**M ± SD**	**M ± SD**	**M ± SD**	**F**	***p***
PANSS^a^	57.86 ± 13.92	49.82 ± 11.09	44.80 ± 10.72	53.11 ± 14.12	48.38 ± 13.99	49.51 ± 16.12	12.51	0.001
Negative PANSS	17.02 ± 7.42	14.39 ± 5.48	12.37 ± 4.75	14.49 ± 6.36	13.36 ± 5.96	13.24 ± 5.99	5.89	0.004
Positive PANSS	11.38 ± 4.03	9.89 ± 3.36	9.11 ± 2.67	10.93 ± 3.95	9.64 ± 3.57	10.47 ± 4.35	6.65	0.002
PSP^b^	59.64 ± 16.29	66.79 ± 12.52	70.71 ± 14.12	64.22 ± 16.17	68.00 ± 19.49	67.11 ± 17.53	3.34	0.039
FBS^c^	18.16 ± 7.55	12.71 ± 6.24	11.48 ± 7.76	16.80 ± 10.39	14.93 ± 10.23	15.40 ± 11.11	5.10	0.008
Family APGAR^d^	6.80 ± 2.42	7.16 ± 2.63	7.55 ± 2.49	6.67 ± 2.52	5.93 ± 3.60	5.73 ± 3.73	4.58	0.013

a *PANSS, Positive and Negative Syndrome Scale (possible scores range from 30 to 210, with higher scores indicating more severe symptoms).*

b *PSP, Personal and Social Performance Scale (possible scores range from 1 to 100, with lower scores indicating worse functioning).*

c *FBS, Family Burden Scale of Disease (possible scores range from 0 to 48, with higher scores indicating more burdens).*

d *Family APGAR, Family APGAR index (possible scores range from 0 to 20, with higher scores indicating a higher level of perceived family function)*.

**Figure 2:**
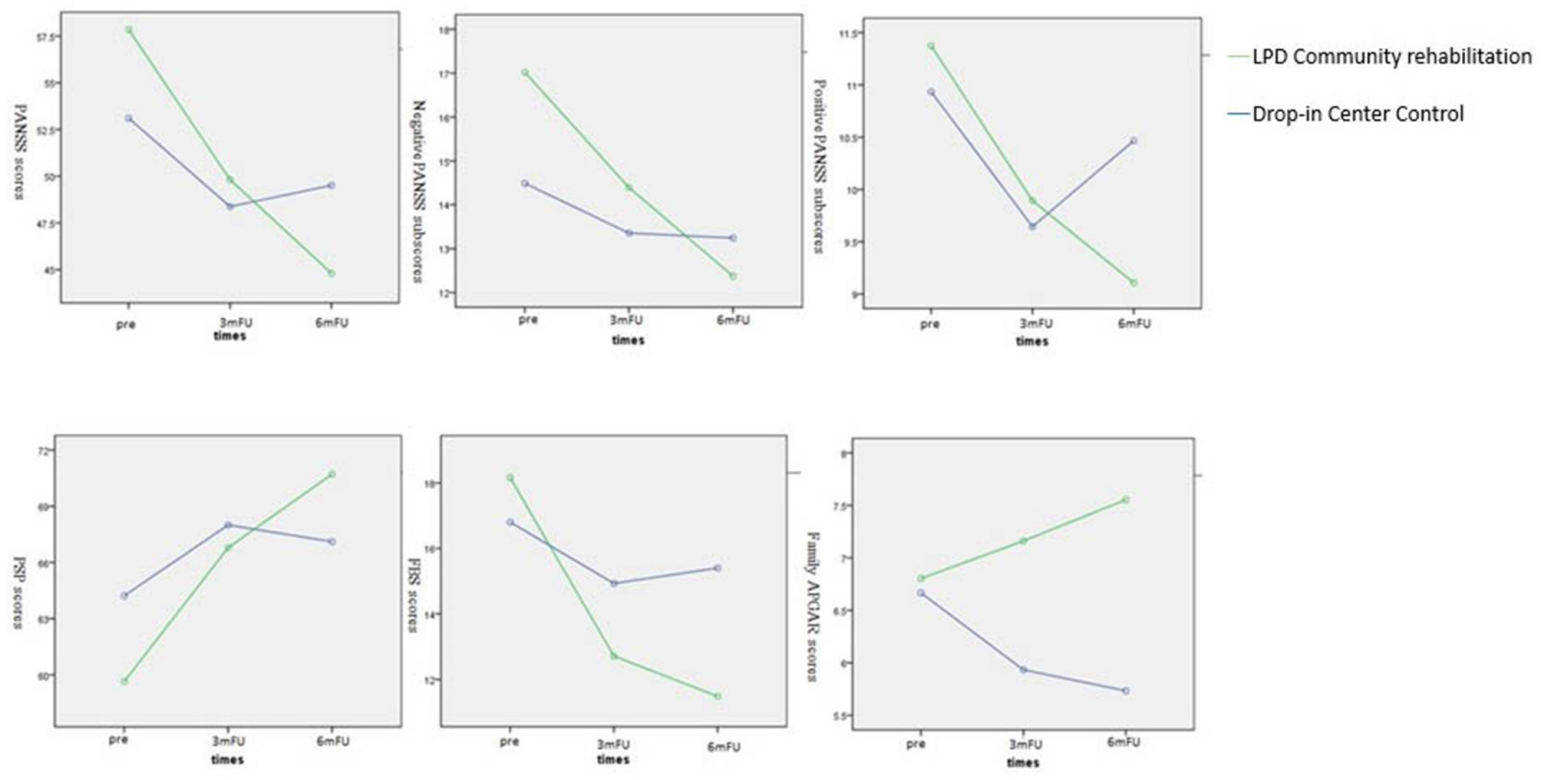
The interaction effects of symptoms, social functioning and family functioning between groups and times. PANSS, Positive and Negative Syndrome Scale; PSP, Personal and Social Performance Scale; FBS, Family Burden Scale of Disease; Family ARGAR, Family ARGAR index; Pre, Pre-intervention; 3mFU, 3-month follow-up; 6mFU, 6-month follow-up; LPD, Lay people delivered.

The means and standard deviations in Table 3 indicate the source of these interactions. The data in the table indicate that the participants' psychiatric symptoms and personal social skills improved significantly more for participants in the LPD psychiatric rehabilitation program than for those who received drop-in center service. Similarly, family care burden and family function improved significantly more for the LPD psychiatric rehabilitation program group than for the drop-in center control group. Figure 2 shows the interaction effects.

## Discussion

The pilot data on the implementation of the LPD community psychiatric rehabilitation program support both its feasibility and efficacy. Changes from baseline to posttreatment and to follow-up indicated significant effects across symptom management, personal social functioning, family care burden, and coherence, with interactions between treatment modality (experimental vs. control groups) and change over time. Our findings support the premise that the LPD community psychiatric rehabilitation program not only provides information but also helps consumers improve their social relationships and cope more effectively with their symptoms. Families also benefit from consumers' participation in the rehabilitation program.

This study found that compared with their control counterparts, persons who participated in the LPD community psychiatric rehabilitation program showed significant improvement in their psychiatric symptoms and social functioning. The specific treatment elements in the LPD program, such as case management, family psychoeducation, and medication management, have been proven effective in significantly reducing psychotic symptomatology in previous studies for both first-episode and chronic populations of schizophrenia (7, 10, 16, 21). These findings may be due to closer monitoring of symptoms in integrated treatment to improve adherence to medication. Social skills training and stress management can improve the ability of patients to cope with stressors, which can help patients cope with their challenges of living in the community (21).

The family of the experimental group also showed less family burden and better family function in adaptability, partnership, growth, affection, and resolution. A previous family psychoeducation program for psychosis patients and their families in six rural towns in China showed that families participating in these programs have a greater understanding of mental illness and less family neglect and abuse (22). Community-based interventions have also been shown to be effective in improving the patients' insight into their treatment and managing their symptoms, which may reduce the number of hospitalizations and reduce the family burden (23).

These pilot data support the feasibility of implementing an LPD rehabilitation program for patients with schizophrenia and the recommendation of the World Health Organization (WHO) that lay nonprofessional health workers deliver community-based psychosocial intervention upgrade services in low-income and developing countries (24). In China, two studies also identified the benefits of LPD for community mental rehabilitation (25, 26). Both studies have their own unique features. One randomized controlled study used mobile text messages for medication reminders, health education, and facilitation of patient care by integrating lay health supporters, village doctors, and psychiatrists while the lay health supporters for case supervision were patient's families or community volunteers, which showed significant improvement in medication adherence and reduction in relapses and re-hospitalizations (25). The other study recruited peers to join the mental health workforce and assist in providing community rehabilitation services, which increased patients' social communication skills and mood (26). Therefore, diverse lay psychiatric service models can be established in different regions according to their own circumstances. A systematic task-share training course with good supervision methods can help implement the service smoothly.

There are several limitations of this study that should be noted. Although the LPD rehabilitation program was delivered in a fixed format, there is no fidelity test to ensure that the curriculum was delivered exactly as it should be. Additionally, the curriculum of the LPD program has not been validated, and thus, critical areas may not have been included in the curriculum. In the control group, the participants voluntarily attended the drop-in center, but their family members did not receive any assistance from the drop-in center, unlike the experimental group families who received family psychoeducation. Thus, the benefit of LPD may be due to the potential Hawthorne effects, because services offered to the LPD group were more intensive. In future research, this study should be replicated with a larger sample size, validation of the curriculum, and a comparable intervention intensity control group. For the outcome assessments, areas such as employment, community functioning, personal empowerment, and sense of purpose should be included rather than merely focusing on symptom management and social functioning.

## Data Availability Statement

The raw data supporting the conclusions of this article will be made available by the authors, without undue reservation.

## Ethics Statement

The studies involving human participants were reviewed and approved by Ethics Committee of West China Hospital of Sichuan University. The patients/participants provided their written informed consent to participate in this study.

## Author Contributions

CL and HD conceptualized and designed the work. YC, HD, EY, and KK collected the data. YC and CL analyzed the data and performed the statistics. YC drafted the manuscript, with critical comments from CL and HD. All authors reviewed the manuscript and approved the final version for submission.

## Conflict of Interest

The authors declare that the research was conducted in the absence of any commercial or financial relationships that could be construed as a potential conflict of interest.

## Publisher's Note

All claims expressed in this article are solely those of the authors and do not necessarily represent those of their affiliated organizations, or those of the publisher, the editors and the reviewers. Any product that may be evaluated in this article, or claim that may be made by its manufacturer, is not guaranteed or endorsed by the publisher.
