# Supplementary material for: The Effectiveness of a Community Psychiatric Rehabilitation Program Led by Laypeople in China: A Randomized Controlled Pilot Study
Source: Front Psychiatry. 2021 Nov 15;12:671217. doi: 10.3389/fpsyt.2021.671217 (PMC8634635; doi:10.3389/fpsyt.2021.671217)
Supplement: Supplementary file 1 [file Data_Sheet_1.docx]

**Supplementary Information**

**Statistical analysis:**

The predictor of gaining benefit from LPD program: The multiple stepwise regression models wereused to identify the predictor of gaining benefit from LPD program. We used the age of onset, duration of illness, number of episode, number of hospitalization, PANSS scores and PSP scores at baseline in LDP group as independent variables and the slopes of PANSS scores and PSP scores from baseline to 6-month follow-up as dependent variables in LDP group.

The results were showed that in LDP group, the more severe of PANSS scores and the longer duration of the illness at baseline, the better improvement of PANSS scores at 6-month follow-up (both p<0.05). And we also found in LDP group, the lower PSP scores, the better improvement of PSP scores at 6-month follow-up (p<0.001).

**Table S1**the stepwise regression models examining relations between the slope of PANSS scores from baseline to 6-month follow-upand sample characteristics at baseline in LDP group

| Variables | Beta | p | 95%CI |
| --- | --- | --- | --- |
| Age of onset | 0.034 | 0.635 | -0.044 to 0.072 |
| Duration of illness | 0.264 | 0.016 | 0.015 to 0.141 |
| Number of episodes | -0.03 | 0.757 | -0.421 to 0.308 |
| Number of hospitalization | -0.086 | 0.462 | -0.393 to 0.181 |
| PANSS scores^a^ | -0.858 | <0.001 | -0.195 to 0.133 |
| PSPscores^b^ | -0.034 | 0.668 | -0.32 to 0.02 |

a. PANSS: Positive and Negative Syndrome Scale (possible scores range from 30 to 210, with higher scores indicating more severe symptoms)

b. PSP: Personal and Social Performance Scale (possible scores range from 1 to 100, with lower scores indicating worse functioning)

**Table S2** the stepwise regression models examining relations between the slope of PANSS scoresfrom baseline to 6-month follow-up and sample characteristics at baseline in LDP group

| Variable | Beta | p | 95%CI |
| --- | --- | --- | --- |
| Age of onset | 0.072 | 0.416 | -0.057 to 0.136 |
| Duration of illness | -0.255 | 0.056 | -0.207 to 0.003 |
| Number of episodes | 0.179 | 0.139 | -0.153 to 1.065 |
| Number of hospitalization | -0.102 | 0.48 | -0.65 to 0.31 |
| PANSSscores^a^ | -0.186 | 0.070 | -0.1 to 0.004 |
| PSPscores^b^ | -0.849 | <0.001 | -0.231 to -0.144 |

a. PANSS: Positive and Negative Syndrome Scale (possible scores range from 30 to 210, with higher scores indicating more severe symptoms)

b. PSP: Personal and Social Performance Scale (possible scores range from 1 to 100, with lower scores indicating worse functioning)

**Table S3**Means (standard deviations) and effect sizes for outcome variables for all participants before and after the intervention

|  | Pre-intervention | 3-month follow-up | 6-month follow-up |  |  |
| --- | --- | --- | --- | --- | --- |
|  | M±SD | M±SD | M±SD | F | *p* |
| Variables |  |  |  |  |  |
| PANSS^a^ | 55.74±14.14 | 49.18±12.42 | 46.9±13.53 | 7.52 | 0.007 |
| Negative PANSS | 15.98±7.05 | 13.93±5.69 | 12.76±5.33 | 0.947 | 0.333 |
| Positive PANSS | 11.18±3.98 | 9.78±3.44 | 9.71±3.56 | 7.96 | 0.006 |
| PSP^b^ | 61.68±16.34 | 67.33±15.93 | 69.11±15.75 | 2.79 | 0.097 |
| FBS^c^ | 17.55±8.91 | 13.7±8.29 | 13.23±9.55 | 6.38 | 0.013 |
| Family ARGAR^d^ | 6.74±2.45 | 6.61±3.14 | 6.74±3.22 | 0.39 | 0.531 |

a. PANSS: Positive and Negative Syndrome Scale (possible scores range from 30 to 210, with higher scores indicating more severe symptoms)

b. PSP: Personal and Social Performance Scale (possible scores range from 1 to 100, with lower scores indicating worse functioning)

c. FBS: Family Burden Scale of Disease (possible scores range from 0 to 48, with higher scores indicating more burdens)

d. Family ARGAR: Family ARGAR index (possible scores range from 0 to 20, with higher scores indicating a higher level of perceived family function)
